# Biosecurity Implications of New Technology and Discovery in Plant Virus Research

**DOI:** 10.1371/journal.ppat.1003337

**Published:** 2013-08-01

**Authors:** Robin MacDiarmid, Brendan Rodoni, Ulrich Melcher, Francisco Ochoa-Corona, Marilyn Roossinck

**Affiliations:** 1 The New Zealand Institute for Plant and Food Research Limited, Auckland, New Zealand; 2 School of Biological Sciences, The University of Auckland, Auckland, New Zealand; 3 Department of Primary Industries, AgriBio – Centre for Agriobioscience, La Trobe University, Bundoora, Victoria, Australia; 4 Department of Biochemistry and Molecular Biology, Oklahoma State University, Stillwater, Oklahoma, United States of America; 5 Institute for Microbial Forensics and Food and Agricultural Biosecurity (NIMFFAB), Department of Entomology and Plant Pathology, Oklahoma State University, Stillwater, Oklahoma, United States of America; 6 Department of Plant Pathology and Environmental Microbiology, Center for Infectious Disease Dynamics, Pennsylvania State University, University Park, Pennsylvania, United States of America; 7 Department of Biology, Center for Infectious Disease Dynamics, Pennsylvania State University, University Park, Pennsylvania, United States of America; The Fox Chase Cancer Center, United States of America

## Abstract

Human activity is causing new encounters between viruses and plants. Anthropogenic interventions include changing land use, decreasing biodiversity, trade, the introduction of new plant and vector species to native landscapes, and changing atmospheric and climatic conditions. The discovery of thousands of new viruses, especially those associated with healthy-appearing native plants, is shifting the paradigm for their role within the ecosystem from foe to friend. The cost of new plant virus incursions can be high and result in the loss of trade and/or production for short or extended periods. We present and justify three recommendations for plant biosecurity to improve communication about plant viruses, assist with the identification of viruses and their impacts, and protect the high economic, social, environmental, and cultural value of our respective nations' unique flora: 1) As part of the burden of proof, countries and jurisdictions should identify what pests already exist in, and which pests pose a risk to, their native flora; 2) Plant virus sequences not associated with a recognized virus infection are designated as “uncultured virus” and tentatively named using the host plant species of greatest known prevalence, the word “virus,” a general location identifier, and a serial number; and 3) Invest in basic research to determine the ecology of known and new viruses with existing and potential new plant hosts and vectors and develop host-virus pathogenicity prediction tools. These recommendations have implications for researchers, risk analysts, biosecurity authorities, and policy makers at both a national and an international level.

## Introduction

Over the last 60 years, global trade has changed significantly with the formation of the World Trade Organization (WTO) in 1995 [1] and the establishment of multiple trade agreements between countries and jurisdictions all aiming at reducing obstacles to international trade. Agreements such as the Sanitary and Phytosanitary (SPS) Agreement have promoted trade liberalization and harmonization of biosecurity measures and agri-food standards but have also facilitated the intracontinental and intercontinental movement of insects and pathogenic microbes that disturb native and exotic ecosystems [2]–[4]. The frequency of incursions of unwanted organisms into these globalized pathways is increasing and leads to emergence of diseases and pests that are costly to control and/or eradicate [5]–[9].

A pest is “any species, strain or biotype of plant, animal or pathogenic agent injurious to plants or plant products,” as defined in a glossary of phytosanitary terms by the International Standards for Phytosanitary Measures (ISPM) [10]. Plant biosecurity is defined as “a set of measures designed to protect crops from emergency plant pests at national, regional and individual farm level” [11], [12]. All WTO countries are required to manage their imports under the SPS Agreement [1], and a core principle of biosecurity policy is founded on risk assessment reflecting scientific evidence and rigorous analysis [13]. Management of pest and disease risk in accordance with internationally accepted principles, as a “no risk” policy, is not possible [14].

At least three parties are involved in international trading transactions: exporters, biosecurity agencies, and importers, but the responsibility for identifying whether new viruses are: 1) present in a country or region and 2) associated with a risk to the local flora is not always clear. It is common for the importing country to adopt a conservative stance and limit the importation of plant material based on the risk of a pathogen that may be associated with the imported material. Therefore, a shared burden of proof exists between trading countries for identifying the biosecurity risk of an organism.

A major implication of plant virus discovery is the impact of new viruses on plant biosecurity. There are at least three distinct motivations for plant virus discovery: 1) identifying causes of viral diseases in crops, 2) screening for specific viruses when their presence is suspected, and 3) sequencing of viruses regardless of symptoms in the target plant or botanical ecosystem. The third and most recent motivation is dependent on the development and implementation of new sequencing technologies resulting in the discovery of new species of viruses and other microorganisms [15]–[21]. Large ecological prospecting projects were initiated in the highly speciose “Area de Conservación Guanacaste” (ACG) in the northwest of Costa Rica [22], the Tallgrass Prairie Preserve (TGP) in Oklahoma, United States [23], and the Great Smokey Mountains National Park, United States [24]. Members of known virus species were only rarely recognized in these metagenomic and ecogenomic surveys, indicating that the discovery of previously unknown plant virus species promises to continue for a considerable length of time [25]. The viruses that are derived from these newly discovered sequences will require names, taxonomic assignments, and epidemiological assessment.

Using deep sequencing technologies has allowed the identification of known and new viruses and provided a new understanding of their biodiversity. In addition to the traditional “pathogenic” role of plant viruses, more intimate symbiotic relationships such as (a) antagonism (one partner benefits at the expense of the other and can manifest in a disease), (b) commensalism (one partner benefits while the other is unaffected), or (c) mutualism (both partners benefit) have been proposed [26]. Examples of conditionally mutualistic relationships were described for several plant viruses and their symptomatic hosts that displayed drought or cold tolerance only when virus-infected [27]. Some plant endogenous pararetroviruses have also been shown to be conditional mutualists, either protecting their host against infection by other related viruses or protecting the plant meristem from virus infection [26]. Despite the potential importance of viruses to their plant hosts, and with the exception of crop plants and some model plants, information about the interaction of most plant species with viruses is not available [23], [28]. Without this plant-virus interaction information, biosecurity risks cannot be defined.

We present and justify three recommendations for plant virus characterization and classification in the context of new virus discovery. These will permit better communication about plant viruses, assist with the identification of viruses and their impacts, and protect the high economic, social, environmental, and cultural value of our respective nations' unique flora.

## Recommendation 1. As Part of the Burden of Proof, Countries and Jurisdictions Should Identify What Pests Already Exist in, and Which Pests Pose a Risk to, Their Native Flora

As the knowledge of virus identity is extended, an equal, if not increased, effort is required to discover viruses' roles in their existing and potential new ecosystems. In particular, since many newly discovered viruses will be derived from infections of native plant hosts, it is vitally important for biosecurity agencies to survey for the presence of plant viruses in their own native flora.

Searches for virus-related sequences in ecogenic and metagenomic discovery projects of natural ecosystems have located many putative viruses. The Lake Needwood project resulted in identification of sequences related to 65 known plant viruses [29], and the ACG project identified about 2,600 distinct viruses (strains or higher taxonomic levels) [22] (Roossinck et al., unpublished). The TGP project identified over 300 distinct viruses with only 18 that corresponded to known viral species. Virus signatures of the major family *Potyviridae* were identified only occasionally in the TGP (Melcher et al., unpublished) by contrast to more than 10% of the infected plants in the ACG [25]. Viruses related to the mycovirus-containing families *Totiviridae*, *Partitiviridae, Chrysoviridae*, and *Endornaviridae* were prevalent in both studies, accounting for about half of the viruses found. Several detected virus sequences proved difficult to classify in specific genera, having properties suggesting their membership in multiple genera or in a novel genus within a family [30].

At the policy level in many countries, risk analyses are applied as decision-making tools that include the identification and assessment of the risk or hazard, the consequences of establishment, and appropriate risk management strategies. Decision-making tools to support pest risk analyses require easy and rapid access to reliable sources of information relating to the plant commodities and native flora at risk. The WTO mandate to demonstrate freedom of a pest as being “known not to occur” within a country or region forms the basis of biosecurity policy for the movement of plant material around the globe. This burden of proof drives the identification of existing and pest plant virus species within a country, including within native flora. For instance, the New Zealand Biosecurity Act [31] was the first national law worldwide to specifically support systematic protection of valued indigenous and introduced biological systems from the harmful effects of exotic pests and diseases. The 1993 act and the associated Biosecurity Strategy for New Zealand [32] state that “Biosecurity is the exclusion, eradication or effective management of risks posed by pests and diseases to the economy, environment and human health.” As a direct result, New Zealand protects plants of high economic, social, environmental, or cultural value, any of which may include native flora. It is critical that countries continue to share the burden of plant biosecurity between national and local governments and industry to facilitate the development of science-based policy on the safe movement of plant material.

For the majority of these virus sequences, it is unclear whether they represent an intact virus (rather than a remnant of a virus expressed from the plant genome) (see Recommendation 2), or whether it has an antagonistic, commensal, and/or a mutualistic relationship with the plant host. More importantly, it is unclear whether the sequenced virus has the capacity to alter that relationship in a new plant host or environment, induce disease symptoms in a new host plant, or recombine with endemic viruses to result in a more virulent or new pathogenic virus species (Recommendation 3).

## Recommendation 2. Plant Virus Sequences Not Associated with a Recognized Virus Infection Are Designated as “Uncultured Virus” and Tentatively Named Using the Host Plant Species of Greatest Known Prevalence, a General Location Identifier, and a Serial Number (e.g., the “Uncultured Virus *Vernonia* TGP1”)

The distinction between a virus as a physical entity and an in-silico virus species that encompasses actual viruses is important when considering naming of viruses [33], [34]. The provision in publications of the italicized name of a virus species indicates that the virus is a true member of a recognized virus species. Conversely, a non-italicized name is usually an invention of the authors of the original publication about the virus and is listed as a tentative virus species by the International Committee for the Taxonomy of Viruses (ICTV).

Naming putative viruses is initially for laboratory convenience. For example, in the TGP study, names comprised an identifying number of the source plant plus the number of the sequence contig generated by sequence assembly, as in 05TGP00101.25 (the 25^th^ contig of the 101^st^ plant sampled in 2005 on the TGP Preserve). Phylogenetic analysis of the retrieved viruslike sequences identifies the most closely related known viral sequences that have been lodged on public databases and determines if the obtained viral sequences are representative of a known or potentially new viral species.

The official naming of a virus occurs when the specific and often unique criteria for virus species definition are reviewed by a panel of experts representing the ICTV [35]. This process is iterative and results in a multiple-year period between discovery of a novel virus signature sequence and the eventual sanctioning of a viral species name. The putative virus has the potential to be considered as a serious international crop threat during this period of deliberation.

The traditional host-disease-virus naming system has limited flexibility to accommodate a large influx of new viruses. Improving naming systems used for “sequence-only” viruses during the laboratory maturation process will assist the ICTV in naming the large numbers of viruses identified using deep sequencing technologies. Based on these experiences and adopted strategies of the TGP project, we suggest the following six criteria for naming of virus sequences, the first three of which have been put forward previously [36].

1. **A name should be associated with the viral sequence rapidly and permanently.** It is not practical to wait for a taxonomic decision before using a name that will be permanently attached to the uncultured virus whose signature has been obtained. Taxonomic revisions should change names as few times as possible, maintaining continuity in the scientific literature and biosecurity documentation.
2. **The names should, in some manner, be informative.** In current practice, informative terms have included: names of the predominant host species, the symptoms associated with infection (see criterion 4), and the location of first isolation (see criterion 3).
3. **The names must be unique and nonambiguous.** It is possible for distinct viral species to have the same host species of original isolation and cause the same disease symptoms. Solutions to naming ambiguity problems have already been implemented by the ICTV. A geographical qualifier can be placed before the “virus” in the name, for example in *Hibiscus latent Singapore virus* (HLSV). Marine bacteriophages are identified by names specifying host, followed by φ and a non-word identifier, such as in *Prochlorococcus marinus* φ P-SSM2 [37]. For some plant viruses, the convention of numbering by adding a unique sequential integer after the name, as in *Grapevine leafroll–associated virus 3*, has been adopted.
4. **The names should not be based on the symptoms caused by infection with the virus.** Recent discoveries of viruses in healthy-appearing plants reveal the folly of using disease symptoms in the name. Not only are many of the new viruses not associated with symptoms in their host of isolation, but the same virus also can cause different symptoms on different plants or plant varieties.
5. **Taxonomic indications should be avoided.** Adding a generic taxonomic prefix to “virus,” for example in “*Tobacco mosaic tobamovirus*” has been presented and discussed [38]–[40], but has not been universally accepted. Premature designation of genus or family status promises to be confusing if it enters the literature. A further difficulty is that the ICTV may change the taxonomic classification of long-classified viruses, necessitating a renaming of viruses with the attendant confusion in the literature.
6. **The naming convention should allow some means of identifying that the virus is a “sequence-only” virus.** The consensus viral genome sequences that result from in-silico assembly and analysis of deep sequencing datasets are not considered acceptable for GenBank/EMBL/DDBJ deposition and are labeled as coming from an “uncultured virus.” It is reasonable therefore that viruses recognized only by their sequence signatures have a lower taxonomic status than fully characterized viruses and adding a “p-,” “e-,” or “s-” prefix, respectively for putative, electronic, or sequence, before the word virus is possible.

## Recommendation 3. Invest in Basic Research to Determine the Ecology of Known and New Viruses with Existing and Potential New Plant Hosts and Vectors and Develop Host-Virus Pathogenicity Prediction Tools

An initial biosecurity investigation might escalate to a full investigation or incursion response when a new or regulated virus, not yet established, is detected within the country's environment. Risk assessment and analysis are the first steps in assessing the economic, social, environmental, or cultural impacts of the new incursion. If the impacts are high, quarantine agencies will assess the various options available. If the likely impacts are low, normally, no further official action is taken. It is therefore important to understand the existing and potential plant virome in our ecosystems. Viruses are part of the wider ecosystem and some interactions may result in disease while other interactions may be beneficial to the host plant.

Some of the more serious plant virus disease epidemics are the result of the introduction of either the host plant or the insect vector into a new region that exposes the crop plant to an endemic or “native” virus, resulting in disease. For example, introduction of cassava into sub-Saharan Africa has seen the emergence of aggressive strains of *Cassava mosaic virus* that have resulted in many farmers abandoning cassava cultivation and consequently destabilizing food security in east Africa [41]. The introduction of the biotype B whitefly, *Bemisia tabaci*, into Brazil in the early 1990s resulted in the horizontal transfer of previously unrecorded indigenous begomoviruses from native onto cultivated plants [42].

Plant viruses range between specialists, with only a single plant host, to generalists that have many hosts. For virus infection to occur, the plant host will lack the defense mechanisms to protect against infection of the invading virus [43]–[45]. Predicting the biological properties of a virus from a gene sequence is an extraordinarily difficult problem requiring a deep understanding of the interaction of the virus with its greater environment [46]. For the foreseeable future, the only reliable predictor of the hazard posed by a virus is empirically based. Opportunities exist for scientific research to provide predictions of pathogenicity of a virus on a specific plant host species (or at least host/non-host family) and for transmissibility by a potential insect vector species (or vector/non-vector family) based on bioinformatics and correlation with biological data. The progressive understanding of plants, their viruses, and the changes that occur within host-virus interactions is therefore vitally important to underpin the development of tools that have the capacity to predict the impact of a virus on a new ecosystem.

## Summary

The intercontinental mobilization of the human population has assisted the spread of infectious diseases on a global scale. This was clearly demonstrated for over half the Australian potyvirus species that have been introduced during the last two centuries by European migrants [47]. Plant virus incursions occur regularly and most are recorded on international databases (e.g., www.promedmail.org). These incursions often result in trade restrictions and the introduction of strict phytosanitary measures on the local industry to prevent further spread and/or to eradicate the pest.

The deep sequencing strategy currently used for plant virus discovery poses significant challenges for biosecurity agencies, particularly with respect to the large number of new plant virus sequences that are being discovered and have the potential to cause disease. Concurrently, there is an increasing number of reports of plant viruses as mutualists, which can provide benefits to the host plant [28], [48]; these mutualistic interactions challenge the traditional dogma that all viruses are pathogens.

In light of these challenges, we have proposed three recommendations to be considered by biosecurity agencies and have identified research gaps that need to be filled.

Globally, communities are demanding improved conservation strategies for native flora and fauna, as demonstrated in the New Zealand Biosecurity Act [31], and it is important that our biosecurity systems meet these expectations. This requires a better understanding of existing fauna and microflora that is present in native ecosystems (Recommendation 1) to improve our ability to identify new and emerging diseases.

Currently, plant quarantine is based on the presence/absence of the pest/pathogen and not the presence of disease that it may cause. As such, in-silico viruses that are being discovered using deep sequencing currently have the potential to disrupt the movement of plant material based on the perception that the virus sequence may cause a disease in a new environment. The second recommendation of this paper is that plant virus sequences that have not been associated with virus infection in a host plant are designated as “uncultured virus” to indicate that the assembled genome sequence is “sequence-only.”

The biological and ecological function of “sequence-only” viruses that are discovered in plants using sequencing strategies is largely unknown [49]. It is clear that many of these viruses are not pathogens and are commensal or mutualists in their hosts. Further study of the ecology of these viruses is required (Recommendation 3) to better understand their relationship with their existing and potential plant hosts.

The cost of plant virus incursions is high and can result in the loss of trade and/or production for extended periods. The three recommendations for plant biosecurity will permit better communication about plant viruses, assist with the identification of viruses and their impacts, facilitate the development of sound biosecurity policy, and protect the high economic, social, environmental, and cultural value of unique flora.
